# Supplementary material for: Impact of The Daily Mile on children’s physical and mental health, and educational attainment in primary schools: iMprOVE cohort study protocol
Source: BMJ Open. 2021 May 28;11(5):e045879. doi: 10.1136/bmjopen-2020-045879 (PMC8166593; doi:10.1136/bmjopen-2020-045879)
Supplement: Supplementary data [file bmjopen-2020-045879supp001.pdf]

Supplemental Table 1. The Daily Mile's ten core principles[1]

Principle		Definition
1.	Quick	Just 15 minutes with no time spent changing, setting up or tidying up. Transitions between class and route should be slick.
2.	Fun	The Daily Mile is physical activity in a social setting and must be fun for the children. They can chat to their friends as they run along enjoying the experience together.
3.	100%	It's always fully inclusive – every child, every day. They should all be out together in the fresh air. Children with mobility difficulties should be supported to take part.
4.	Weather	Treat the weather as a benefit, not a barrier. Children enjoy being outside in the different types of weather, connecting with nature and being aware of the seasons.
5.	Route	Ideally, your Daily Mile route should have a firm and mud-free surface – most schools use the playground or an existing path. Incorporating child-pleasing loops and squiggles works well.
6.	Risk	Risk assess the route in order to ensure The Daily Mile is a safe activity.
7.	When to Go	The Daily Mile should happen during curricular time, at least 3 times a week. Ideally, the class teacher should decide when to go out – they know their class and can respond flexibly to their needs.
8.	Clothes	The children run in their school clothes without changing into kit, putting jackets on if it's cold or damp and taking sweatshirts off if it's warm.
9.	Own Pace	The children go at their own pace. Done properly, it's not a walk – able-bodied children should aim to run or jog for the full 15 minutes with only occasional stops to catch their breath, if necessary.
10.	Simple	Keep it simple. Resist the temptation to over complicate it. It should always be social and fun. From time-to-time, you may wish to connect it to the curriculum or do something seasonal, for example, running 'Laps to Lapland'.

[1]*The Daily Mile Foundation (https://thedailymile.co.uk/steps-to-success/)*
