# Supplementary material for: Impact of The Daily Mile on children’s physical and mental health, and educational attainment in primary schools: iMprOVE cohort study protocol
Source: BMJ Open. 2021 May 28;11(5):e045879. doi: 10.1136/bmjopen-2020-045879 (PMC8166593; doi:10.1136/bmjopen-2020-045879)
Supplement: Supplementary data [file bmjopen-2020-045879supp002.pdf]

Supplemental Table 2. Outcomes and measuring tools included in the iMprOVE study

Outcome	Measured by	Outcome/completed by
Physical activity	• Device-based (accelerometer)	Children's physical activity
	• Children's Physical Activity Questionnaire (C-PAQ)[a] Parent	Parent/Carer to complete about their child's physical activity
	• International Physical Activity Questionnaire (IPAQ)[b]	Parent/Carer's* physical activity
Mental health	• The Warwick and Edinburgh Mental Wellbeing Scale (WEMWBS)[c]	Parent/Carer's* mental health
	• Strengths and Difficulties Questionnaire (SDQ)[e]	Parent/Carer's* and Teachers to complete about the child
Educational attainment	• Likert scale from 1 (below expected levels) to 5 (above expected levels)[f]	Teacher's ratings
General health and wellbeing	• Child Health Utility 9D (CHU-9D)[d]	Parent/Carers to complete for their children in school years 1 and 2. For children in years 3 to 6, children will be encouraged to complete questions by themselves with help from parent/carer.
	• EuroQol Visual Analogue Scale (EuroQol VAS)[g]	Parent/Carer's general health, and parent/carer to complete for their child in school years 1 and 2. Children in years 3 to 6, children will be encouraged to complete questions by themselves with help from parent/carer.
	• EuroQol Descriptive Scale[g]	Parent/Carer general health
	• Questions from the UK Census 2011[h]	Parent/Carer general health, and parent/carer to complete for their child in school years 1 and 2. Children in years 3 to 6, children will be encouraged to complete questions by themselves with help from parent/carer.

	• Life satisfaction, worthwhile, happiness and anxiety questions from Measuring National Wellbeing (MNW)[i]	Parent/Carer wellbeing
Socio-demographic	• Questions from the UK Census 2011[h]	Parent/Carer's own socio-demographic data, and parent/carer to complete socio-demographic data about their child if their child is in school years 1 or 2. Children in years 3 to 5 will be encouraged to complete these questions on their own with help from their parent/career.
Anthropometry	• Height measure<br>• Bioimpedance scales	Children's height and weight.

*Any parent/carer for the child can complete the questionnaires at baseline. For consistency, we will ask the parent/carer who completed the questionnaire at baseline to complete questionnaires at each follow-up.

[a]Kowalski k, Crocker R and Donen R. The Physical Activity Questionnaire for Older Children (PAQ-C) and Adolescents (PAQ-Q) Manual. University of Saskatchewan.

[b]Craig, C. L., et al. (2003). "International physical activity questionnaire: 12-country reliability and validity." Med Sci Sports Exerc 35: 1381-95.

[c]Tenant R, Hiller L, Fishwick R et al. The Warwick-Edinburgh Mental Well-being Scale (WEMWBS): development and UK validation. *Health Qual Life Outcomes.* 2007;5. DOI: 10.1186/1477-7525-5-63.

[d]Stevens KJ. Working with Children to Develop Dimensions for a Preference-Based, Generic, Paediatric, Health-Related Quality-of-Life Measure. *Qual Health Res.* 2010;20(3):340-351. DOI: 10.1177/1049732309358328.

[e]Goodman R. The Strengths and Difficulties Questionnaire: a research note. *Journal of Child Psychology and Psychiatry.* 1997;38(5):581-586. DOI: 10.1111/j.1469-7610.1997.tb01545.x.

[f]Breheny K, Passmore S, Adab P, et al. Effectiveness and cost-effectiveness of The Daily Mile on childhood weight outcomes and wellbeing: a cluster randomised controlled trial. *International Journal of Obesity.* 2020;44(4):812-822. DOI: 10.1038/s41366-019-0511-0.

[g]EuroQol Research Foundation. EQ-5D-5L User Guide 2019.

[h]UK Census United Kingdom. Office for National Statistics (ONS). 2011.

[i]Measuring National Wellbeing – Personal Wellbeing. Office for National Statistics, 2019
