# Supplementary material for: Impact of The Daily Mile on children’s physical and mental health, and educational attainment in primary schools: iMprOVE cohort study protocol
Source: BMJ Open. 2021 May 28;11(5):e045879. doi: 10.1136/bmjopen-2020-045879 (PMC8166593; doi:10.1136/bmjopen-2020-045879)
Supplement: Supplementary data [file bmjopen-2020-045879supp003.pdf]

Supplemental Table 3. iMprOVE study sample size including clustering at school level, Intraclass Correlation (ICC) of 0.05, and 60% attrition

Unit (cluster) level	School
Clusters	77
Cluster size (pupils)	46
Allocation ratio	1:1
Control group MVPA (mins)	53
Intervention group MVPA (mins)	58.5
Variance MVPA	484
Standard deviation MVPA	22
Power	0.9
Alpha (two-sided)	0.05
ICC[1]	0.05
Total sample size (without inflation)	2208
Drop out	60
Extra pupils	1325
Total sample size (inflated for drop-out)	3533
**Sample size per group**	**1766**

[1]ICC: Intraclass correlation
