# Supplementary material for: Impact of The Daily Mile on children’s physical and mental health, and educational attainment in primary schools: iMprOVE cohort study protocol
Source: BMJ Open. 2021 May 28;11(5):e045879. doi: 10.1136/bmjopen-2020-045879 (PMC8166593; doi:10.1136/bmjopen-2020-045879)
Supplement: Author's manuscript [file bmjopen-2020-045879.draft_revisions.pdf]

BMJ Open is committed to open peer review. As part of this commitment we make the peer review history of every article we publish publicly available.

When an article is published we post the peer reviewers' comments and the authors' responses online. We also post the versions of the paper that were used during peer review. These are the versions that the peer review comments apply to.

The versions of the paper that follow are the versions that were submitted during the peer review process. They are not the versions of record or the final published versions. They should not be cited or distributed as the published version of this manuscript.

BMJ Open is an open access journal and the full, final, typeset and author-corrected version of record of the manuscript is available on our site with no access controls, subscription charges or pay-per-view fees (http://bmjopen.bmj.com).

If you have any questions on BMJ Open's open peer review process please email info.bmjopen@bmj.com

BMJ Open

Impact of The Daily Mile™ on children's physical and mental health and educational attainment in primary schools; iMprOVE cohort study protocol

Journal:	*BMJ Open*
Manuscript ID	bmjopen-2020-045879
Article Type:	Protocol
Date Submitted by the Author:	18-Oct-2020

Complete List of Authors:	Ram, Bina; Imperial College London Faculty of Medicine, School of Public Health<br>Chalkley, Anna; Loughborough University, School of Sport, Exercise and Health Sciences<br>van Sluijs, Esther; Cambridge University, MRC Epidemiology Unit<br>Phillips, Rachel; Imperial College London Faculty of Medicine, Faculty of Medicine, School of Public Health<br>Venkatraman, Tishya; Imperial College London Faculty of Medicine, School of Public Health<br>Hargreaves, Dougal; Imperial College London Faculty of Medicine, School of Public Health<br>Viner, Russell; University College London Institute of Child Health, Population, policy and practice research programme<br>Saxena, Sonia; Imperial College London, School of Public Health

Keywords:	PUBLIC HEALTH, EPIDEMIOLOGY, Community child health < PAEDIATRICS

SCHOLARONE™
Manuscripts

I, the Submitting Author has the right to grant and does grant on behalf of all authors of the Work (as defined in the below author licence), an exclusive licence and/or a non-exclusive licence for contributions from authors who are: i) UK Crown employees; ii) where BMJ has agreed a CC-BY licence shall apply, and/or iii) in accordance with the terms applicable for US Federal Government officers or employees acting as part of their official duties; on a worldwide, perpetual, irrevocable, royalty-free basis to BMJ Publishing Group Ltd ("BMJ") its licensees and where the relevant Journal is co-owned by BMJ to the co-owners of the Journal, to publish the Work in this journal and any other BMJ products and to exploit all rights, as set out in our licence.

The Submitting Author accepts and understands that any supply made under these terms is made by BMJ to the Submitting Author unless you are acting as an employee on behalf of your employer or a postgraduate student of an affiliated institution which is paying any applicable article publishing charge ("APC") for Open Access articles. Where the Submitting Author wishes to make the Work available on an Open Access basis (and intends to pay the relevant APC), the terms of reuse of such Open Access shall be governed by a Creative Commons licence – details of these licences and which Creative Commons licence will apply to this Work are set out in our licence referred to above.

Other than as permitted in any relevant BMJ Author's Self Archiving Policies, I confirm this Work has not been accepted for publication elsewhere, is not being considered for publication elsewhere and does not duplicate material already published. I confirm all authors consent to publication of this Work and authorise the granting of this licence.

For peer review only

Impact of The Daily Mile™ on children's physical and mental health and educational attainment in primary schools; iMprOVE cohort study protocol

Bina Ram[1], Anna Chalkley[2], Esther M F van Sluijs[3], Rachel Phillips[1], Tishya Venkatraman[1], Dougal S Hargreaves[4], Russell Viner[5], Sonia Saxena[1]

[1]School of Public Health, Imperial College London, UK
[2]School of Sport, Exercise and Health Sciences, Loughborough University, UK
[3]Centre for Diet and Activity Research & MRC Epidemiology Unit, School of Clinical Medicine, University of Cambridge, UK
[4]Department for Education, UK
[5] Institute of Child Health, University College London, UK

Corresponding author

Dr Bina Ram
School of Public Health
Department of Primary Care and Public Health
Imperial College London
The Reynolds Building, Charing Cross Campus
St Dunstan's Road
London
W6 8RP

Email: b.ram@imperial.ac.uk

ORCiD

Bina Ram	0000-0003-0023-1573
Anna Chalkley	0000-0002-1163-6210
Esther M F van Sluijs	0000-0001-9141-9082
Rachel Phillips	0000-0002-3634-7845
Tishya Venkatraman	0000-0001-6171-2384
Dougal S Hargreaves	0000-0003-0722-9847
Russell Viner	0000-0003-3047-2247
Sonia Saxena	0000-0003-3787-2083

For peer review only - http://bmjopen.bmj.com/site/about/guidelines.xhtml

37 Target journal: BMJ Open

38 Manuscript word count: 3167/4000 words

For peer review only

For peer review only - http://bmjopen.bmj.com/site/about/guidelines.xhtml

Abstract

Introduction

School-based active mile initiatives such as The Daily Mile™ are widely promoted to address shortfalls in meeting physical activity recommendations. The iMprOVE study aims to examine the impact of The Daily Mile on children's physical and mental health and educational attainment throughout primary school.

Methods and analysis

iMprOVE is a longitudinal quasi-experimental cohort study. We will send a survey to all state-funded primary schools in Greater London to identify participation in The Daily Mile for non-random allocation to the intervention (Daily Mile schools) and control (non-Daily Mile schools) groups from those that respond. We aim to recruit 3,533 Year 1 children (aged 5-6 years) from 77 primary schools for baseline measures and follow them up annually until the end of primary school. Data collection includes objectively measured moderate to vigorous physical activity (MVPA), mental health assessed using the Strengths and Difficulties Questionnaire, and educational attainment (ratings from 'below expected' to 'above expected levels'). The primary outcome is the mean change in MVPA minutes from baseline to Year 6 during the school day among the intervention group compared with controls. We will use multilevel linear regression models adjusting for socio-demographic data and participation in The Daily Mile. The study is powered to detect a 10% (5.5 minute) difference between the intervention and control group which would be considered clinically significant.

Ethics and dissemination

Ethics has been approved from Imperial College Research Ethics Committee, reference 20IC6127. Key findings will be disseminated to the public through research networks, social, print and media broadcasts, community engagement opportunities and schools. We will work with policy makers for direct application and impact of our findings.

Keywords

The Daily Mile, active mile initiatives, physical activity, primary schools, mental health

For peer review only - http://bmjopen.bmj.com/site/about/guidelines.xhtml

Article summary

Strengths and limitations of this study

- This quasi-experimental study is the most comprehensive to date to examine the longitudinal impact of The Daily Mile on children's health.
- Including schools in Greater London will reflect the diverse multi-ethnic primary school population.
- Low response rates from school surveys may result in a biased sample.
- Attrition may affect precision of effect sizes and bias results.

For peer review only

For peer review only - http://bmjopen.bmj.com/site/about/guidelines.xhtml

Introduction

Increasing physical activity during childhood is a global goal to improve health in the population and reduce the growing health risks from non-communicable diseases.[1, 2] Regular physical activity is associated with better cardiorespiratory and muscular fitness and bone health.[3] A growing science base suggests links between children's physical activity and psychological and social health including sleep, cognition, self-confidence and social interaction.[3] This is particularly important in childhood where health behaviours may become embedded.[4] The United Kingdom's (UK) Chief Medical Officers recommend that children and young people, aged 5 to 18 years, should engage in sport and physical activity for an average of 60 minutes or more every day.[5, 6] The latest national self-report activity figures for children and young people by Sport England, showed that around 47% are meeting these targets in the UK whilst 29% participate in less than an average of 30 minutes a day.[5]

School-based active mile initiatives are widely publicised to address significant shortfalls in children's physical activity.[7] Schools are considered ideal settings for physical activity initiatives given their wide reach.[8, 9] The Daily Mile™ (TDM) is a school-based active mile initiative that has been promoted in the UK's Childhood Obesity Strategy as an easy and accessible way to increase children's physical activity during the school day to improve their fitness, health and wellbeing.[10] TDM was launched in 2012 in one primary school in Scotland, UK. Now, 1 in 5 schools in England are registered with TDM[11] and it has a growing global adoption.[12] It is a teacher-led, free and simple initiative that involves children running or jogging for 15 minutes ('a mile') at least three times a week in addition to national curriculum physical education and scheduled break times. As schooling is compulsory for all children in England, school-based active mile initiatives and have the potential to level health inequalities among children by reaching those living in poverty.[13]

TDM appears to be reaching a nationally representative primary school population. Up to August 2019, 22% of all state-funded primary schools in England were registered, reaching a population of just over 963,000 children.[14] The evolving evidence base of the health benefits of TDM is promising; children in TDM schools have demonstrated improved fitness from 12 weeks to 12 months of taking up this initiative.[15, 16] However, there is limited evidence of its impact on children's mental health[17, 18] and it is only shown to be broadly associated with higher academic performance.[19] Furthermore, some of the previous studies have focused on weight-related outcomes such as body fat and body mass index (BMI) and have reported conflicting results.[16, 18] TDM was not designed as an obesity prevention initiative and there is inconclusive evidence that increased physical activity alone is effective in preventing obesity in children.[20]

A major gap in the evidence is the lack of data; there is limited evidence on how TDM is implemented in schools outside of experimental studies and limited follow-up times.[21-23] Currently, there are no evaluations of the impact and sustainability of TDM throughout primary school life.

The iMprOVE study will provide a robust real-world evaluation of the impact of TDM on children's health, wellbeing and educational attainment over the course of primary school.

Aims and research questions

The aim of the iMprOVE study is to compare the change in moderate to vigorous physical activity (MVPA) of children in TDM schools with non-TDM schools across primary school years.

For peer review only - http://bmjopen.bmj.com/site/about/guidelines.xhtml

The following research questions will be addressed:

- What is the difference in MVPA accumulated during the school day between children in TDM and non-TDM schools across primary school years?
- What are the differences in mental health and educational attainment of children in TDM and non-TDM schools across primary school years?
- To what extent do primary schools in Greater London implement TDM according to its core principles?
- Are there any differential impacts of TDM on health in sub-groups of children, schools and areas with greater health need?

Methods and analysis

Study design

The iMprOVE study is a longitudinal quasi-experimental cohort study of state-funded primary schools located in Greater London which has a diverse multi-ethnic urban population. The target population is children in schools that participate in TDM (intervention group) compared with those that do not participate in TDM or any other active mile initiative (control group). We will assess children in the first year of primary school (aged 5 to 6 years) and follow them up annually in each successive primary school year until the end of primary school (Year 6, children aged 10 to 11 years). In total, we will collect six waves of data (Figure 1).

The intervention

TDM involves every child running or jogging at their own pace for 15 minutes, at least three times a week during the school day, in addition to curricular lessons including physical education (PE) and scheduled break times. There are ten core principles for successful delivery of TDM which schools are encouraged to follow (Supplemental Table 1). These include that TDM should be conducted in all weathers, and the full 15 minutes is spent on the activity without having to change clothing or shoes.[24] TDM is also flexible to local adaptation by schools.

Since 2017, we have held several professional and public multi-stakeholder consultations about which of the ten core principles of TDM were essential criteria for successful implementation. The consultations included health care professionals, researchers and members from The Daily Mile Foundation. A consensus was reached agreeing a definition for school participation in TDM which was that three essential principles were adhered to: (1) the whole school takes part; (2) children are active (i.e., running or jogging) for 15 minutes; and (3) it is implemented at least three times a week.

Procedure

School survey and recruitment

We will send a school survey to headteachers in all state-funded primary schools in Greater London (n=1721) derived from our database of primary schools in England.[25] Responses from the survey will identify whether schools participate in TDM and if they do, how many of the ten principles are adhered to. Schools which participate in the three essential principles of TDM identified from the public and

For peer review only - http://bmjopen.bmj.com/site/about/guidelines.xhtml

expert consultation will be assigned to the intervention group, and schools not participating in TDM or any other active mile initiative will be assigned to the control group. Primary schools where TDM is not implemented by the whole school will be considered ineligible for the study. For example, if they report only some classes or year groups take part, thus meeting only two of the three criteria considered essential (Figure 2). We will contact the eligible schools to participate in the next stage of the study where through the schools, we will recruit year 1 primary school children (aged 5 to 6 years) and their parent/carer by letter and information sheet. For a child to be eligible to take part, their parent/carer will also need to agree to participate.

Informed consent

Written and informed consent will be sought at the point of recruitment, prior to any data collection. We will first obtain consent from the headteacher of the school to allow the distribution of invitation letters to parents. Parents who agree to participate in the study will be emailed a consent form (for their participation), a parental consent form (allowing their child to participate) and a child assent form. Consent to take part in the study at baseline (Wave 1) will assume contact for subsequent follow-ups. At each follow-up, we will provide an option for participants to decline further participation.

Outcomes and measures

We will collect data from children, parents and teachers which are detailed in Supplemental Table 2. The main outcome is children's mean MVPA minutes during the school day (assuming a start time of 9am and end time of 3pm) measured using wrist-worn accelerometers over a consecutive 7-day wear period. To capture all MVPA and consider compensatory effects, we all also assess minutes of MVPA outside school hours on weekdays and total minutes of MVPA during weekends.

The Strengths and Difficulties Questionnaire (SDQ), a measure of child mental health,[26] is a brief behavioural screening questionnaire about children and young people aged from 2 years to 17 years. The SDQ has five sub-scales each including 5 items that assess emotional symptoms, conduct problems, hyperactivity, peer relationship problems, and prosocial behaviour. It has been extensively validated[27] and is one of the most widely and internationally used measures for assessing children's mental health.[28]

Educational attainment will be measured by teacher ratings of the child's reading, writing and math ability on a 5-point Likert-scale ranging from 1 (below expected levels) to 5 (above expected levels).[18] Ratings will be based on the UK's National Curriculum's 'age related expectations' which are the standard expectations defined by threshold descriptors indicating what pupils should be able to achieve by the end of primary school.[29]

Secondary outcomes

We will assess children's self-reported (completed by proxy) intensity of physical activity using the Children's Physical Activity Questionnaire (C-PAQ).[30] Collecting self-reported physical activity alongside accelerometer data will enable us to validate our objective measure of physical activity.

The Child Health Utility 9D (CHU-9D) will measure children's health related quality of life (HRQoL).[31] The CHU-9D assesses how children are feeling 'today' by addressing 9 items (worry, sadness, pain, tiredness, annoyance, problems with schoolwork, their daily routine, and their ability to join in with activities). It is suitable for children aged from 7 years to 11 years and can be completed by proxy for

For peer review only - http://bmjopen.bmj.com/site/about/guidelines.xhtml

younger children.[32] The CHU-9D has good psychometric performance and shows potential as a valid and reliable instrument for measuring HRQoL.[33-35]

We will measure children's height and weight from which we will calculate each child's body mass index (BMI) as weight/height^2 (kg/m^2) and standardized by age and sex.

Covariates

Parental physical and mental health is a strong predictor of child functioning.[36] We will therefore collect parental self-reported physical activity, mental health, wellbeing and general health. The International Physical Activity Questionnaire (IPAQ) – Short Form[37] will measure time spent in different intensities of physical activity during the last 7 days. We will use the Warwick and Edinburgh Mental Wellbeing Scale (WEMWBS) which includes fourteen items of feeling and functioning aspects of wellbeing to measure parental mental health.[38]

We will collect socio-demographic data on children's date of birth, sex, and ethnic group, and parental socio-demographic data including age, sex, ethnic group, highest level of qualification and employment status.

School characteristics including school size (i.e., overall pupil numbers), class size, ethnic composition, area level of deprivation and number of pupils eligible for free school meals will be obtained from publicly available data provided by the Department for Education. School participation in TDM will be sought at the start of each follow-up data collection.

Data collection

We will email a link to the questionnaires to parent/carers and class teachers. Paper versions will be available on request. Parents will complete the child questionnaires by proxy.

The iMprOVE research team will arrange a convenient time to visit the school to take anthropometry measures of the children and issue and fit them with the accelerometer. Verbal instructions of wearing the monitor will be given along with an information pack including details of wearing the monitor correctly, how long to wear, and when and how to return the monitors. Incentives (shopping voucher) will be offered to the child to encourage successful completion of all their corresponding data collection.

All measures taken at baseline will be repeated at each follow-up. From Wave 3 onwards, the children (now in Year Group 3 and aged 7 to 8 years) will be encouraged to complete their own questionnaires with help from their parent/carer. Incentives for the children after each wave of data collection will be offered to encourage families to continue taking part.

Data collection time points

There will be one data collection in each school year. Data collection for baseline (Wave 1) will begin from January 2021 (allowing for regular TDM activity to take place if implemented by the school) and will continue until we have met our sample size targets. Control group participants will be assessed simultaneously to the intervention group (Figure 1).

For consistency and potential seasonal variation in activity levels, we aim to carry out follow-up assessments during the same month as baseline assessments in each year until the end of data collection in 2026 (Figure 1).

For peer review only - http://bmjopen.bmj.com/site/about/guidelines.xhtml

Sample size

Our sample size is based on the primary outcome of mean MVPA (minutes) per day allowing for the clustering of children in the same school and the primary analysis adjusting for baseline minutes of MVPA. In Greater London, there is an average of 46 children per state-funded primary school in Year 1.[25]

A total sample of 3,533 children (approximately 1,767 children in the intervention and control groups) across 77 schools gives the study 90% power to detect a 5.5 minute increase in the mean daily average MVPA minutes between children in TDM schools compared with non-TDM schools, assuming a mean daily average MVPA of 53 minutes (standard deviation 22) in the control group based on the study by Chesham et al.[16] This has been inflated to allow for 60% attrition across the follow-up period and assumes a two-sided alpha equal to 0.05. To allow for the analyses to include clustering at school level, we have applied an intraclass correlation coefficient of 0.05 to our calculation (Supplemental Table 3). An increase of just over 5 minutes of MVPA per day equal to a 10% difference would be clinically significant to increase physical activity among the proportion of children who participate in less than an average of 30 minutes per day.

Statistical analyses

For every wave of data collection, the main analysis will treat the outcomes as continuous variables. This will enable us to understand the impact of TDM in any change observed in physical activity, mental health and educational attainment for each child through each successive year of primary school.

We will use a linear multilevel mixed effect regression model to estimate the mean difference in MVPA values between TDM schools and non-TDM schools across follow-up and at each follow-up wave. Children will be included as a random intercept with fixed effects for wave, intervention group, wave-by-intervention group interaction and baseline (Wave 1) MVPA. School will also be included in the model as a random effect and adjustments for covariates including participation in TDM and socio-demographic variables will be included. Baseline adjusted group differences will be reported with a 95% confidence interval for each wave. An additional model without wave-by-intervention interaction will be fitted to provide an estimate of the overall intervention effect across follow-up.

Based on previous studies,[36, 39] we expect data from children to vary throughout each wave of data collection as compliance in the youngest age groups (5 to 6 years) may differ compared with when they are measured later (aged 10 to 11 years). Decisions on valid accelerometer data to include in the analysis will be made when reviewing the data and based on previous literature.

Linear multilevel mixed effect regression models as described for MVPA will be repeated for the outcomes of mental health and educational attainment.

Sub-group analysis

To address inequalities, we will examine MVPA of children in TDM and non-TDM schools according to area level deprivation (a measure of poverty) using the Index of Multiple Deprivation[40].

For peer review only - http://bmjopen.bmj.com/site/about/guidelines.xhtml

Sensitivity analyses

We anticipate that during the study period, schools that are assigned to intervention group may stop TDM, and schools assigned to the control group may take up TDM. We will conduct a series of sensitivity analyses to explore TDM impacts in children who engage in TDM in some year groups only. We will also consider seasonal effects on implementation of TDM and MVPA overall dependent on the month children are assessed. In addition, missing data for MVPA in the follow-up waves will be imputed and included separately to a complete case analysis.

All analyses will be carried out using STATA v15 (StataCorp, Texas, USA).

Patient and public involvement

We have consulted professional and public representatives within the management structure (The Daily Mile Research Advisory Group including members from The Daily Mile Foundation, academic researchers, and representatives from Sport England, London Marathon and London Sport, and Patient and Public Involvement including parents and children who have been approached for piloting questionnaires and assessments) throughout the design of the study and who will continue to be consulted throughout the duration of the study. Advice on the design of study materials (e.g., study information sheets and questionnaires), participant engagement, and how to retain participants in the study will be sought throughout the study period. Questionnaires will be piloted with parents and children through workshops.

Discussion

Currently, physical activity has fallen to lowest rates following the Covid-19 pandemic lockdowns and restrictions. Global policies are focusing on safe ways that will allow children to adapt to primary school life that allows improved health behaviours aimed at increasing physical activity. Active mile initiatives in school settings are seen as part of the solution to rebuilding and embedding health behaviours in early life.

The iMprOVE study will be the most comprehensive evaluation of an active mile initiative and the first longitudinal study to capture the impact of participation over primary school life.

Key strengths of the iMprOVE study are its size, the assessment of an intervention in a real world setting, and individual level data and longitudinal follow-up throughout primary school life. Furthermore, the objective measure of physical activity using accelerometers will provide more accurate estimates of daily minutes of MVPA than parent-reported physical activity in questionnaires, minimising potential participant bias of overestimating or underestimating self-reported time spent being active.[41] The inclusion of schools from a diverse multi-ethnic population and urban setting enables us to examine differential impacts of TDM among different socio-demographic groups. However, there are a number of important limitations to consider. Since iMprOVE is a quasi-experimental cohort study and not randomised, there is likely to be an unequal number of participants in the intervention and control groups. We therefore expect some selection bias in schools that agree to participate in the study and as with previous cohort studies, there may be underrepresentation of school populations from disadvantaged areas.

For peer review only - http://bmjopen.bmj.com/site/about/guidelines.xhtml

Given the popularity of TDM and its widescale adoption across the UK and globally, the results of this study will be of interest to parents, teachers and public health policy makers. The findings will determine the potential of TDM and other similar active mile initiatives, to improve the health, wellbeing and educational attainment for all children in primary schools and inform national strategies to reduce inequalities in child health.

Ethics and dissemination

Full ethical approval has been granted by Imperial College Research Ethics Committee (ICREC), reference 20IC6127.

Wider dissemination of key findings to the public will be through research networks, social (Facebook, Twitter), print (newspapers) and media broadcasts (news outlets). We will engage with study participants through community engagement activities, presentations, and newsletters to schools, teachers, parents and children taking part in the study. We will work with policy makers nationally to ensure direct application and impact of our work.

For peer review only

Project steering

The management structure of this study includes the following groups:

1. Investigators (Principal Investigator and Lead Researcher)
2. Research support team (collaborators)
3. Project steering group (advisors)

Author contributions

SS wrote the original application and obtained funding for the study. SS and BR conceptualized the cohort study. BR, SS, AC, TV, EvS and DH designed the study. BR wrote and methodology and developed all the study materials. BR and SS wrote the ethics application and gained ethical approval from Imperial College Research Ethics Committee (ICREC). BR produced the first draft of this manuscript. All authors contributed to redrafts.

Funding statement

This work is supported by The Daily Mile Foundation supported by INEOS.

BR is funded by The Daily Mile Foundation. SS holds grants from The Daily Mile Foundation and is funded by the National Institute for Health Research (NIHR) School for Public Health Research (SPHR) and the NIHR North West London Applied Research Collaboration (NW London ARC). TV is funded by an NIHR SPHR PhD Studentship (Grant Reference Number PD-SPH-2015-10055). DH is part of the NIHR SPHR and NW London ARC, and funded by the Department for Education (DfE).

EvS is supported by the Medical Research Council (MRC) (Grant MC_UU_12015/7). The work by EvS is undertaken under the auspices of the Centre for Diet and Activity Research (CEDAR), where funding from Cancer Research UK, the British Heart Foundation, the Economic and Social Research Council, the Medical Research Council, the National Institute for Health Research, and the Wellcome Trust, under the auspices of the UK Clinical Research Collaboration, is gratefully acknowledged (087636/Z/08/Z; ES/G0007462/1; MR/K023187/1).

Acknowledgements

We are grateful for support from the NIHR School for Public Health Research and the NIHR North West NW London ARC.

The NIHR School for Public Health Research is a partnership between the Universities of: Sheffield; Bristol; Cambridge; Imperial; and University College London; The London School for Hygiene and Tropical Medicine (LSHTM); LiLaC – a collaboration between the Universities of Liverpool and Lancaster; and Fuse - The Centre for Translational Research in Public Health a collaboration between Newcastle, Durham, Northumbria, Sunderland and Teesside Universities.

The Department of Primary Care and Public Health is grateful for support from the NIHR Biomedical Research Centre funding scheme, the NIHR School for Public Health Research, and the NIHR North West London Applied Research Collaboration.

We thank The Daily Mile Foundation for funding and support.

For peer review only - http://bmjopen.bmj.com/site/about/guidelines.xhtml

The views expressed here are those of the authors and not necessarily those of the NIHR, the Department of Health and Social Care, the NHS, DfE or The Daily Mile Foundation.

Competing interests

SS and BR received funding from The Daily Mile Foundation. SS, BR, AC and TV are members of The Daily Mile Research Advisory Group.

Patient consent for publication

Not required.

Data sharing statement

Data sharing is not applicable to this protocol. Data collected as part of this research will be available to external researchers on completion of the study, and proposals for collaboration prior to, during and after study completion are welcome.

Open access

This is an open access article distributed in accordance with the Creative Commons Attribution Non Commercial (CC BY-NC 4.0) license, which permits others to distribute, remix, adapt, build upon this work non-commercially, and license their derivative works on different terms, provided the original work is properly cited, appropriate credit is given, any changes made indicated, and the use is non-commercial. See: http:// creativecommons. org/ licenses/ by- nc/ 4. 0/.

Figures

Figure 1 iMprOVE study design.

Figure 2 Identifying schools to be included in the iMprOVE study.

For peer review only

References

1. WHO. Global recommendations on physical activity for health. Geneva: World Health Organisation; 2010.

2. WHO. Global Health Risks: Mortality and burden of disease attributable to selected major risks. Geneva: World Health Organisation; 2009.

3. World Health Organisation. Global Strategy on Diet, Physical Activity and Health; Physical activity and young people Geneva2011 [Available from: https://www.who.int/dietphysicalactivity/factsheet_young_people/en/ [Accessed Jul 2020].

4. NCMP. National Child Measurement Programme (NCMP), England 2018/19 School Year. United Kingdom: National Statistics, NHS Digital; 2019.

5. Sport England. Active Lives: Children and Young People Survey, Academic Year 2018/19. UK; 2019.

6. Department of Health and Social Care. Physical activity guidelines: UK Chief Medical Officers' Report. GOV.UK; 2019.

7. Public Health England. Active mile briefing: evidence and policy summary. United Kingdom: Loughborough University, UK, for Public Health England; 2020.

8. Waters E, de Silva-Sanigorski A, Hall BJ, et al. Interventions for preventing obesity in children. Cochrane Database Syst Rev. 2011(12):CD001871.

9. Wang Y, Cai L, Wu Y, et al. What childhood obesity prevention programmes work? A systematic review and meta-analysis. Obes Rev. 2015;16(7):547-65.

10. HM Government. Childhood obesity: a plan for action Chapter 2. United Kingdom; 2018.

11. The Daily Mile Foundation. The Daily Mile [Available from: https://thedailymile.co.uk/.

12. The Daily Mile UK. The Daily Mile Global Map United Kingdom2019 [Available from: https://thedailymile.co.uk/participation-map/ [Accessed March 2020].

13. Marmot M, Allen J, Boyce T, et al. Health equity in England: The Marmot Review 10 years on. London: Institute of Health Equity; 2020.

For peer review only - http://bmjopen.bmj.com/site/about/guidelines.xhtml

14. Foley KA, Venkatraman T, Ram B, et al. Protocol for developing a core outcome set for evaluating school-based physical activity interventions in primary schools. BMJ Open. 2019;9(12):e031868.

15. Marchant E, Todd C, Stratton G, et al. The Daily Mile: Whole-school recommendations for implementation and sustainability. A mixed-methods study. PLoS One. 2020;15(2):e0228149.

16. Chesham RA, Booth JN, Sweeney EL, et al. The Daily Mile makes primary school children more active, less sedentary and improves their fitness and body composition: a quasi-experimental pilot study. BMC Medicine. 2018;16(1):64.

17. Morris JL, Daly-Smith A, Archbold VS, et al. The Daily Mile (TM) initiative: Exploring physical activity and the acute effects on executive function and academic performance in primary school children. Psychol Sport Exerc. 2019;45.

18. Breheny K, Passmore S, Adab P, et al. Effectiveness and cost-effectiveness of The Daily Mile on childhood weight outcomes and wellbeing: a cluster randomised controlled trial. Int J Obesity. 2020;44(4):812-22.

19. Booth JN, Chesham RA, Brooks NE, et al. A citizen science study of short physical activity breaks at school: improvements in cognition and wellbeing with self-paced activity. BMC Med. 2020;18(1):62.

20. Wareham NJ, van Sluijs EM, Ekelund U. Physical activity and obesity prevention: a review of the current evidence. Proc Nutr Soc. 2005;64(2):229-47.

21. Hanckel B, Ruta D, Scott G, et al. The Daily Mile as a public health intervention: a rapid ethnographic assessment of uptake and implementation in South London, UK. BMC Public Health. 2019;19(1):1167.

22. Malden S, Doi L. The Daily Mile: teachers' perspectives of the barriers and facilitators to the delivery of a school-based physical activity intervention. BMJ Open. 2019;9(3):e027169.

23. Ryde GC, Booth JN, Brooks NE, et al. The Daily Mile: What factors are associated with its implementation success? PLoS One. 2018;13(10):e0204988.

24. The Daily Mile UK. The Daily Mile Core Principles United Kingdom2019 [Available from: https://thedailymile.co.uk/steps-to-success [Accessed March 2020].

25. Venkatraman T, Honeyford K, Costelloe C, et al. Sociodemographic profiles, educational attainment and physical activity associated with The Daily Mile registration in primary schools in England – a national cross-sectional linkage study. Journal of Epidemiology and Community Health. 2020(0):1-8.

For peer review only - http://bmjopen.bmj.com/site/about/guidelines.xhtml

26. Goodman R. The Strengths and Difficulties Questionnaire: a research note. J Child Psychol Psychiatry. 1997;38(5):581-6.

27. Muris P, Meesters C, van den Berg F. The Strengths and Difficulties Questionnaire (SDQ)--further evidence for its reliability and validity in a community sample of Dutch children and adolescents. Eur Child Adolesc Psychiatry. 2003;12(1):1-8.

28. CORC. Strengths and Difficulties Questionnaire (SDQ) London, UK: Child Outcomes Research Consortium; 2002 [Available from: https://www.corc.uk.net/outcome-experience-measures/strengths-and-difficulties-questionnaire/ [Accessed Jul 2019].

29. NFER. Understanding age-related expectations UK: The National Foundation for Educational Research in England and Wales; 2002-2018 [Available from: https://www.nfer.ac.uk/for-schools/free-resources-advice/assessment-hub/introduction-to-assessment/understanding-age-related-expectations/ [Accessed Jul 2020].

30. Kowalski K, Crocker R, Donen R. The Physical Activity Questionnaire for Older Children (PAQ-C) and Adolescents (PAQ-Q) Manual. Canada: University of Saskatchewan; 2004.

31. Stevens KJ. Working with children to develop dimensions for a preference-based, generic, pediatric, health-related quality-of-life measure. Qual Health Res. 2010;20(3):340-51.

32. Stevens K. CHU-9D: Measuring health and calculating QALYs for children and adolescents UK: The University of Sheffield; 2016 [Available from: https://licensing.sheffield.ac.uk/i/health-outcomes/CHU-9D.html [Accessed Jan 2020].

33. Stevens K. Valuation of the Child Health Utility 9D Index. Pharmacoeconomics. 2012;30(8):729-47.

34. Stevens K, Ratcliffe J. Measuring and valuing health benefits for economic evaluation in adolescence: an assessment of the practicality and validity of the child health utility 9D in the Australian adolescent population. Value Health. 2012;15(8):1092-9.

35. Petersen KD, Ratcliffe J, Chen G, et al. The construct validity of the Child Health Utility 9D-DK instrument. Health Qual Life Outcomes. 2019;17(1):187.

36. Jago R, Solomon-Moore E, Toumpakari Z, et al. Parents' perspectives of change in child physical activity & screen-viewing between Y1 (5-6) & Y4 (8-9) of primary school: implications for behaviour change. BMC Public Health. 2018;18(1):520.

37. Craig CL, Marshall AL, Sjostrom M, et al. International physical activity questionnaire: 12-country reliability and validity. Med Sci Sports Exerc. 2003;35(8):1381-95.

For peer review only

For peer review only - http://bmjopen.bmj.com/site/about/guidelines.xhtml

38. Tennant R, Hiller L, Fishwick R, et al. The Warwick-Edinburgh Mental Well-being Scale (WEMWBS): development and UK validation. Health Qual Life Outcomes. 2007;5:63.

39. Jago R, Salway R, Emm-Collison L, et al. Association of BMI category with change in children's physical activity between ages 6 and 11 years: a longitudinal study. Int J Obes (Lond). 2020;44(1):104-13.

40. Ministry of Housing CaLG. English Indices of Multiple Deprivation London, UK: Natiional Statistics; 2019 [Available from: https://www.gov.uk/government/statistics/english-indices-of-deprivation-2019 [Accessed June 2020].

41. DAPA measurement toolkit. [Available from: https://dapa-toolkit.mrc.ac.uk.

For peer review only

For peer review only - http://bmjopen.bmj.com/site/about/guidelines.xhtml

Figure 1. iMprOVE study design

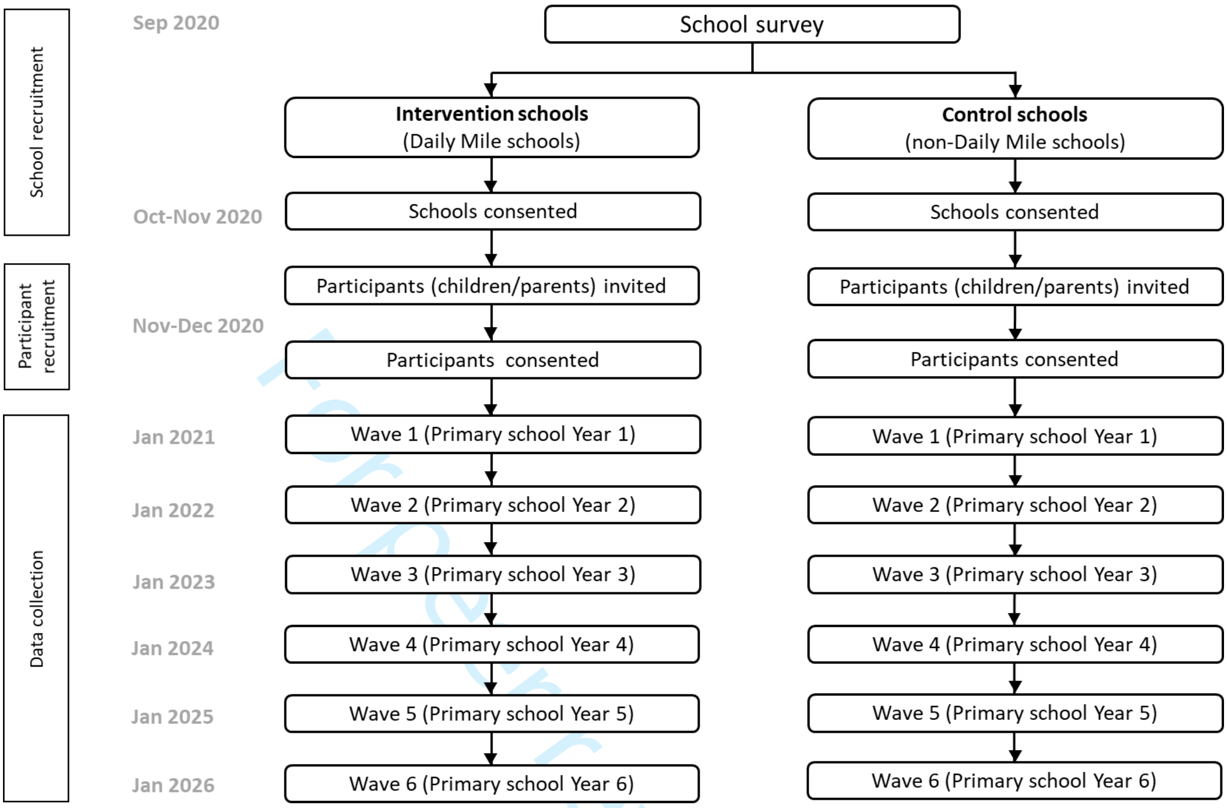

For peer review only

Figure 2. Identifying schools to be included in the iMprOVE study

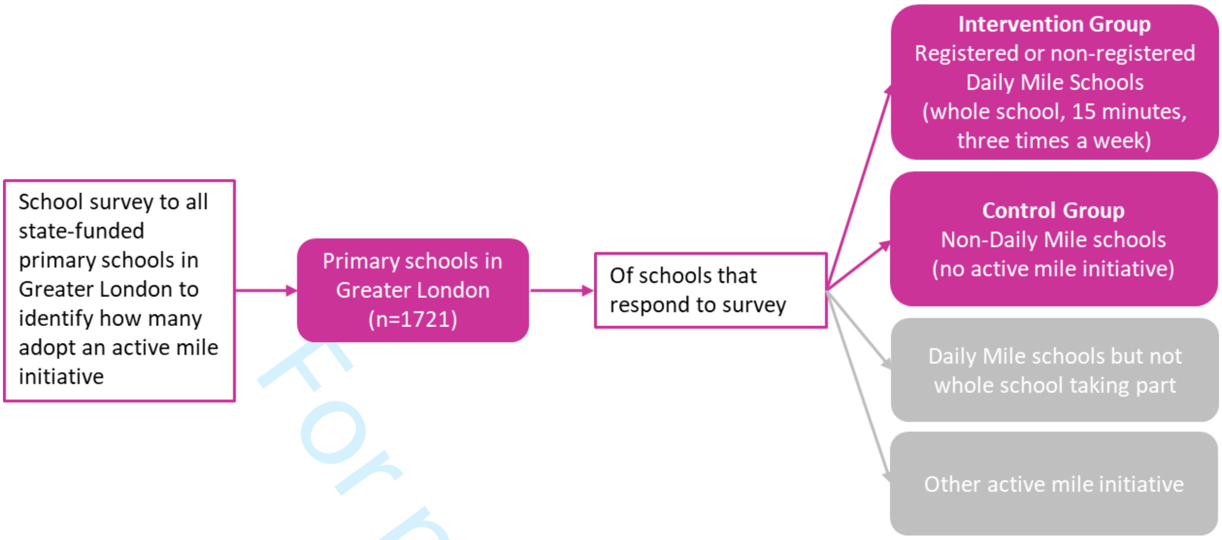

For peer review only

Supplemental Table 1. The Daily Mile's ten core principles[1]

Principle	Definition
1. Quick	Just 15 minutes with no time spent changing, setting up or tidying up. Transitions between class and route should be slick.
2. Fun	The Daily Mile is physical activity in a social setting and must be fun for the children. They can chat to their friends as they run along enjoying the experience together.
3. 100%	It's always fully inclusive – every child, every day. They should all be out together in the fresh air. Children with mobility difficulties should be supported to take part.
4. Weather	Treat the weather as a benefit, not a barrier. Children enjoy being outside in the different types of weather, connecting with nature and being aware of the seasons.
5. Route	Ideally, your Daily Mile route should have a firm and mud-free surface – most schools use the playground or an existing path. Incorporating child-pleasing loops and squiggles works well.
6. Risk	Risk assess the route in order to ensure The Daily Mile is a safe activity.
7. When to Go	The Daily Mile should happen during curricular time, at least 3 times a week. Ideally, the class teacher should decide when to go out – they know their class and can respond flexibly to their needs.
8. Clothes	The children run in their school clothes without changing into kit, putting jackets on if it's cold or damp and taking sweatshirts off if it's warm.
9. Own Pace	The children go at their own pace. Done properly, it's not a walk – able-bodied children should aim to run or jog for the full 15 minutes with only occasional stops to catch their breath, if necessary.
10. Simple	Keep it simple. Resist the temptation to over complicate it. It should always be social and fun. From time-to-time, you may wish to connect it to the curriculum or do something seasonal, for example, running 'Laps to Lapland'.

[1]*The Daily Mile Foundation (https://thedailymile.co.uk/steps-to-success/)*

For peer review only

Supplemental Table 2. Outcomes and measuring tools included in the iMprOVE study

Outcome	Measured by	Completed by
Physical activity	• Device-based (accelerometer)<br>• Children's Physical Activity Questionnaire (C-PAQ)[a] Parent<br>• International Physical Activity Questionnaire (IPAQ)[b]	Child<br>Parent, Child[†]<br>Parent
Mental health	• The Warwick and Edinburgh Mental Wellbeing Scale (WEMWBS)[c]<br>• Strengths and Difficulties Questionnaire (SDQ)[e]	Parent<br>Parent, Teacher
Educational attainment	• Likert scale from 1 (below expected levels) to 5 (above expected levels)[f]	Teacher
General health and wellbeing	• Child Health Utility 9D (CHU-9D)[d]<br>• EuroQol Visual Analogue Scale (EuroQol VAS)[g]<br>• EuroQol Descriptive Scale[g]<br>• Questions from the UK Census 2011[h]<br>• Life satisfaction, worthwhile, happiness and anxiety questions from Measuring National Wellbeing (MNW)[i]	Parent, Child[†]<br>Parent, Child[†]<br>Parent<br>Parent, Child[†]<br>Parent
Socio-demographic	• Questions from the UK Census 2011[h]	Parent, Child[†]
Anthropometry	• Height measure<br>• Bioimpedance scales	Child

[†]Completed by proxy by parent/carer for child in school year groups 1 and 2. Children from years 3 to 6 will be encouraged to complete questionnaires with help from parent/carer
[a]Kowalski k, Crocker R and Donen R. The Physical Activity Questionnaire for Older Children (PAQ-C) and Adolescents (PAQ-Q) Manual. University of Saskatchewan.
[b]Craig, C. L., et al. (2003). "International physical activity questionnaire: 12-country reliability and validity." Med Sci Sports Exerc 35: 1381-95.
[c]Tenant R, Hiller L, Fishwick R et al. The Warwick-Edinburgh Mental Well-being Scale (WEMWBS): development and UK validation. *Health Qual Life Outcomes.* 2007;5. DOI: 10.1186/1477-7525-5-63.
[d]Stevens KJ. Working with Children to Develop Dimensions for a Preference-Based, Generic, Paediatric, Health-Related Quality-of-Life Measure. *Qual Health Res.* 2010;20(3):340-351. DOI: 10.1177/1049732309358328.
[e]Goodman R. The Strengths and Difficulties Questionnaire: a research note. *Journal of Child Psychology and Psychiatry.* 1997;38(5):581-586. DOI: 10.1111/j.1469-7610.1997.tb01545.x.
[f]Breheny K, Passmore S, Adab P, et al. Effectiveness and cost-effectiveness of The Daily Mile on childhood weight outcomes and wellbeing: a cluster randomised controlled trial. *International Journal of Obesity.* 2020;44(4):812-822. DOI: 10.1038/s41366-019-0511-0.
[g]EuroQol Research Foundation. EQ-5D-5L User Guide 2019.
[h]UK Census United Kingdom. Office for National Statistics (ONS). 2011.
[i]Measuring National Wellbeing – Personal Wellbeing. Office for National Statistics, 2019

For peer review only

Supplemental Table 3. iMprOVE study sample size including clustering at school level, Intraclass Correlation (ICC) of 0.05, and 60% attrition

Unit (cluster) level	School
Clusters	77
Cluster size (pupils)	46
Allocation ratio	1:1
Control group MVPA (mins)	53
Intervention group MVPA (mins)	58.5
Variance MVPA	484
Standard deviation MVPA	22
Power	0.9
Alpha (two-sided)	0.05
ICC[1]	0.05
Total sample size (without inflation)	2208
Drop out	60
Extra pupils	1325
Total sample size (inflated for drop-out)	3533
**Sample size per group**	**1766**

[1]ICC: Intraclass correlation

For peer review only

BMJ Open

Impact of The Daily Mile™ on children's physical and mental health and educational attainment in primary schools; iMprOVE cohort study protocol

Journal:	*BMJ Open*
Manuscript ID	bmjopen-2020-045879.R1
Article Type:	Protocol
Date Submitted by the Author:	15-Mar-2021
Complete List of Authors:	Ram, Bina; Imperial College London Faculty of Medicine, School of Public Health<br>Chalkley, Anna; Loughborough University, School of Sport, Exercise and Health Sciences<br>van Sluijs, Esther; Cambridge University, MRC Epidemiology Unit<br>Phillips, Rachel; Imperial College London Faculty of Medicine, Faculty of Medicine, School of Public Health<br>Venkatraman, Tishya; Imperial College London Faculty of Medicine, School of Public Health<br>Hargreaves, Dougal; Imperial College London Faculty of Medicine, School of Public Health<br>Viner, Russell; University College London Institute of Child Health, Population, policy and practice research programme<br>Saxena, Sonia; Imperial College London, School of Public Health
**Primary Subject Heading**:	Public health
Secondary Subject Heading:	Epidemiology
Keywords:	PUBLIC HEALTH, EPIDEMIOLOGY, Community child health < PAEDIATRICS

SCHOLARONE™
Manuscripts

I, the Submitting Author has the right to grant and does grant on behalf of all authors of the Work (as defined in the below author licence), an exclusive licence and/or a non-exclusive licence for contributions from authors who are: i) UK Crown employees; ii) where BMJ has agreed a CC-BY licence shall apply, and/or iii) in accordance with the terms applicable for US Federal Government officers or employees acting as part of their official duties; on a worldwide, perpetual, irrevocable, royalty-free basis to BMJ Publishing Group Ltd ("BMJ") its licensees and where the relevant Journal is co-owned by BMJ to the co-owners of the Journal, to publish the Work in this journal and any other BMJ products and to exploit all rights, as set out in our licence.

The Submitting Author accepts and understands that any supply made under these terms is made by BMJ to the Submitting Author unless you are acting as an employee on behalf of your employer or a postgraduate student of an affiliated institution which is paying any applicable article publishing charge ("APC") for Open Access articles. Where the Submitting Author wishes to make the Work available on an Open Access basis (and intends to pay the relevant APC), the terms of reuse of such Open Access shall be governed by a Creative Commons licence – details of these licences and which Creative Commons licence will apply to this Work are set out in our licence referred to above.

Other than as permitted in any relevant BMJ Author's Self Archiving Policies, I confirm this Work has not been accepted for publication elsewhere, is not being considered for publication elsewhere and does not duplicate material already published. I confirm all authors consent to publication of this Work and authorise the granting of this licence.

For peer review only

Impact of The Daily Mile™ on children's physical and mental health and educational attainment in primary schools; iMprOVE cohort study protocol

Bina Ram[1], Anna Chalkley[2], Esther M F van Sluijs[3], Rachel Phillips[1], Tishya Venkatraman[1], Dougal S Hargreaves[1,4], Russell Viner[5], Sonia Saxena[1]

[1]School of Public Health, Imperial College London, UK
[2]School of Sport, Exercise and Health Sciences, Loughborough University, UK
[3]Centre for Diet and Activity Research & MRC Epidemiology Unit, School of Clinical Medicine, University of Cambridge, UK
[4]Department for Education, UK
[5] Institute of Child Health, University College London, UK

Corresponding author

Dr Bina Ram
School of Public Health
Department of Primary Care and Public Health
Imperial College London
The Reynolds Building, Charing Cross Campus
St Dunstan's Road
London
W6 8RP

Email: b.ram@imperial.ac.uk

ORCiD

Bina Ram	0000-0003-0023-1573
Anna Chalkley	0000-0002-1163-6210
Esther M F van Sluijs	0000-0001-9141-9082
Rachel Phillips	0000-0002-3634-7845
Tishya Venkatraman	0000-0001-6171-2384
Dougal S Hargreaves	0000-0003-0722-9847
Russell Viner	0000-0003-3047-2247
Sonia Saxena	0000-0003-3787-2083

For peer review only

For peer review only - http://bmjopen.bmj.com/site/about/guidelines.xhtml

Target journal: BMJ Open

Manuscript word count: 3742/4000 words

For peer review only

For peer review only - http://bmjopen.bmj.com/site/about/guidelines.xhtml

Abstract

Introduction

School-based active mile initiatives such as The Daily Mile™ are widely promoted to address shortfalls in meeting physical activity recommendations. The iMprOVE study aims to examine the impact of The Daily Mile on children's physical and mental health and educational attainment throughout primary school.

Methods and analysis

iMprOVE is a longitudinal quasi-experimental cohort study. We will send a survey to all state-funded primary schools in Greater London to identify participation in The Daily Mile. The survey responses will be used for non-random allocation to either the intervention group (Daily Mile schools), or to the control group (non-Daily Mile schools). We aim to recruit 3,533 Year 1 children (aged 5-6 years) from 77 primary schools and follow them up annually until the end of their primary school years. Data collection taking place at baseline (children in school Year 1) and each primary school year thereafter, includes device based measures of moderate to vigorous physical activity (MVPA), and questionnaires to measure mental health (Strengths and Difficulties Questionnaire), and educational attainment (ratings from 'below expected' to 'above expected levels'). The primary outcome is the mean change in MVPA minutes from baseline to Year 6 during the school day among the intervention group compared with controls. We will use multilevel linear regression models adjusting for socio-demographic data and participation in The Daily Mile. The study is powered to detect a 10% (5.5 minute) difference between the intervention and control group which would be considered clinically significant.

Ethics and dissemination

Ethics has been approved from Imperial College Research Ethics Committee, reference 20IC6127. Key findings will be disseminated to the public through research networks, social, print and media broadcasts, community engagement opportunities and schools. We will work with policy makers for direct application and impact of our findings.

Keywords

The Daily Mile, active mile initiatives, physical activity, primary schools, mental health

For peer review only - http://bmjopen.bmj.com/site/about/guidelines.xhtml

Article summary

Strengths and limitations of this study

- This quasi-experimental study is the most comprehensive to date to examine the longitudinal impact of The Daily Mile on children's health.
- Including schools in Greater London will reflect the diverse multi-ethnic primary school population.
- Low response rates from school surveys may result in a biased sample.
- Attrition may affect precision of effect sizes and bias results.

For peer review only

For peer review only - http://bmjopen.bmj.com/site/about/guidelines.xhtml

Introduction

Increasing physical activity during childhood is a global goal to improve health in the population and reduce the growing health risks from non-communicable diseases.[1, 2] Regular physical activity is associated with better cardiorespiratory and muscular fitness and bone health.[3] A growing science base suggests links between children's physical activity and psychological and social health including sleep, cognition, self-confidence and social interaction.[3] This is particularly important in childhood where health behaviours may become embedded.[4] The United Kingdom's (UK) Chief Medical Officers recommend that children and young people, aged 5 to 18 years, should engage in sport and physical activity for an average of 60 minutes or more every day.[5] The latest national self-report activity figures for children and young people by Sport England, showed that around 47% are meeting these targets in the UK whilst 29% participate in less than an average of 30 minutes a day.[6]

School-based active mile initiatives are widely publicised to address significant shortfalls in children's physical activity.[7] Schools are considered ideal settings for physical activity initiatives given their wide reach.[8, 9] The Daily Mile™ (TDM) is a school-based active mile initiative that was launched in 2012 by a head teacher in Scotland, UK, who wanted to increase the fitness of her pupils.[10] Since 2012, 1 in 5 schools in England are now registered with TDM [11] and it has a growing global adoption.[12] TDM is a teacher-led, free and simple initiative that involves children running or jogging for 15 minutes ('a mile') at least three times a week, in addition to national curriculum physical education and scheduled break times. TDM has been promoted in the UK's Childhood Obesity Plan as an easy and accessible way to increase children's physical activity during the school day to improve their fitness, health and wellbeing.[13] As schooling is compulsory for all children in England, school-based active mile initiatives have the potential to reach all children, including those living in poverty, which may help to reduce the gap in physical activity among children.[14]

TDM appears to be reaching a nationally representative primary school population. Up to August 2019, 22% of all state-funded primary schools in England were registered, reaching a population of just over 963,000 children.[11] The evolving evidence base of the benefits of TDM on physical activity is promising; a study by Chesham et al (2018) showed an increase in children's moderate to vigorous physical activity (MVPA) by ~9 minutes,[15] and a study by Morris et al (2020) demonstrated that during one active mile lesson, children accumulated between 5 and 15 minutes of MVPA.[16] Whilst there are acute assessments suggesting that children accumulate around an average of 10 minutes of MVPA during TDM, there is limited evidence of its impact on children's mental health [17, 18] and inconsistencies on benefits to academic performance.[16, 19] Furthermore, some of the previous studies have focused on weight-related outcomes such as body fat and body mass index (BMI) and have reported conflicting results.[15, 20] TDM was not designed as an obesity prevention initiative and there is inconclusive evidence that increased physical activity alone is effective in preventing obesity in children.[21] The evidence base still lacks an assessment of the impact and sustainability of TDM throughout primary school life.

A major gap in the evidence is the lack of data; there is limited evidence on how TDM is implemented in schools outside of experimental studies and limited follow-up times.[22-24] Currently, there are no evaluations of the impact and sustainability of TDM throughout primary school life.

For peer review only - http://bmjopen.bmj.com/site/about/guidelines.xhtml

The iMprOVE study will provide a robust real-world evaluation of the impact of TDM on children's health, wellbeing and educational attainment over the course of primary school.

Aims and research questions

The aim of the iMprOVE study is to compare the change in moderate to vigorous physical activity (MVPA) of children in TDM schools with non-TDM schools across primary school years.

The following research questions will be addressed:

- What is the difference in MVPA accumulated during the school day between children in TDM and non-TDM schools across primary school years?
- What are the differences in mental health and educational attainment of children in TDM and non-TDM schools across primary school years?
- How much MVPA is attributed to doing TDM and how much does this contribute to children's overall physical activity?
- To what extent do primary schools in Greater London implement TDM according to its core principles?
- Are there any differential impacts of TDM on health in sub-groups of children, schools and areas with greater health need?

Methods and analysis

Study design

The iMprOVE study is a longitudinal quasi-experimental cohort study of state-funded primary schools located in Greater London which has a diverse multi-ethnic urban population. The target population is children in schools that participate in TDM (intervention group) compared with those that do not participate in TDM or any other active mile initiative (control group). We will assess children in the first year of primary school (aged 5 to 6 years) and follow them up annually in each successive primary school year until the end of primary school (Year 6, children aged 10 to 11 years). In total, we will collect six waves of data (Figure 1).

The intervention

TDM involves every child running or jogging at their own pace for 15 minutes, at least three times a week during the school day., TDM is in addition to curricular lessons including physical education (PE) and scheduled break times. There are ten core principles for successful delivery of TDM which schools are encouraged to follow (Supplemental Table 1). These include that TDM should be conducted in all weathers, and the full 15 minutes is spent on the activity without having to change clothing or shoes.[25] TDM is also flexible to local adaptation by schools.

Since 2017, we have held several professional and public multi-stakeholder consultations about which of the ten core principles of TDM were essential criteria for successful implementation. The consultations included health care professionals, researchers and members from TDM Foundation. A consensus was reached agreeing a definition for school participation in TDM which was that three essential principles

For peer review only - http://bmjopen.bmj.com/site/about/guidelines.xhtml

were adhered to: (1) the whole school takes part; (2) children are running or jogging for 15 minutes; and (3) it is implemented at least three times a week.

Procedure

School survey and recruitment

To identify which schools to assign to the intervention and control groups at baseline, we will send a school survey by post (including a link to the online version) with a freepost return envelope to all state-funded primary schools in Greater London (n=1721) derived from our database of primary schools in England.[11] Any teacher who is best placed to answer questions about their school's involvement with TDM can complete the survey. Schools that do not respond will be contacted by email two weeks after initial contact with a further email one week later. If required, we will conduct one further follow-up by phone to encourage survey completion two weeks after the second email. Responses from the survey will identify whether schools participate in TDM and if they do, how many of the ten principles are adhered to. Schools which participate in the three essential principles of TDM identified from the public and expert consultation will be assigned to the intervention group, and schools not participating in TDM or any other active mile initiative will be assigned to the control group. Primary schools where TDM is not implemented by the whole school will be considered ineligible for the study. For example, if they report only some classes or year groups take part, thus meeting only two of the three criteria considered essential (Figure 2). We will contact the eligible schools to participate in the next stage of the study where through the schools, we will recruit year 1 primary school children (aged 5 to 6 years) and their parent/carer by letter and information sheet. For a child to be eligible to take part, their parent/carer will also need to agree to participate.

Informed consent

Written and informed consent will be sought at the point of recruitment, prior to any data collection. We will first obtain consent from the headteacher of the school to allow the distribution of invitation letters to parents. Parents who agree to participate in the study will be emailed a consent form (for their participation), a parental consent form (allowing their child to participate) and a child assent form. Consent to take part in the study at baseline (Wave 1) will assume contact for subsequent follow-ups. At each follow-up, we will provide an option for participants to decline further participation.

Outcomes and measures

We will collect data from children, parents and teachers which are detailed in Supplemental Table 2. The main outcome is children's mean MVPA minutes during the school day (we will match individual children to the start and end times of their school as reported by the teacher) measured using the GENEActiv (Activinsights, Cambridge, UK) wrist-worn accelerometers. The GENEActiv accelerometer is worn 24/7 including when in water and during sleep. Children will be instructed to wear the monitors for 24 hours on each day of a consecutive 7-day wear period. The accelerometers will be initialised for 9 days to obtain 7 full days of wear (excluding the date of issue and day of collecting the accelerometers). We will use raw acceleration data to classify wear time and assess time spent in activity intensities (including moderate, vigorous and moderate to vigorous physical activity). The threshold for identifying non-wear time will be based on reviewing the acceleration data and will follow a process to correct for diurnal bias similar to that used in previous studies.[26, 27] As the use and analysis of wrist-worn accelerometer data in younger age groups is still developing,[28, 29] we will review and adapt our

For peer review only - http://bmjopen.bmj.com/site/about/guidelines.xhtml

process to reflect the updated knowledge and best practice at the time of first analysis. We will use segmented analysis to identify MVPA during TDM activities as reported by the class teacher, and to consider compensatory effects, we all assess minutes of MVPA during school hours and outside school hours, and on weekdays overall and at the weekend.

The Strengths and Difficulties Questionnaire (SDQ), a measure of child mental health,[30] is a brief behavioural screening questionnaire about children and young people aged from 2 years to 17 years. The SDQ has five sub-scales each including 5 items that assess emotional symptoms, conduct problems, hyperactivity, peer relationship problems, and prosocial behaviour. It has been extensively validated[31] and is one of the most widely and internationally used measures for assessing children's mental health.[32] Parent/Carers and teachers will complete the SDQ about the child.

Educational attainment will be measured by teacher ratings of the child's reading, writing and math ability on a 5-point Likert-scale ranging from 1 (below expected levels) to 5 (above expected levels).[20] Ratings will be based on the UK's National Curriculum's 'age related expectations' which are the standard expectations defined by threshold descriptors indicating what pupils should be able to achieve by the end of primary school.[33]

Secondary outcomes

We will assess children's self-reported (or completed by proxy) intensity of physical activity using the Children's Physical Activity Questionnaire (C-PAQ).[34] Collecting self-reported physical activity alongside accelerometer data will enable us to validate self-reported data with our objective measure of physical activity.

The Child Health Utility 9D (CHU-9D) will measure children's health related quality of life (HRQoL).[35] The CHU-9D assesses how children are feeling 'today' by addressing 9 items (worry, sadness, pain, tiredness, annoyance, problems with schoolwork, their daily routine, and their ability to join in with activities). It is suitable for children aged from 7 years to 11 years and can be completed by proxy for younger children.[36] The CHU-9D has good psychometric performance and shows potential as a valid and reliable instrument for measuring HRQoL.[37-39]

At baseline (children in school year 1) and at the first follow-up (children in year 2) the C-PAQ and CHU-9D will be completed by proxy about the child by the parent/carer. From school year 3 until the end of primary school, children will be encouraged to complete their own questionnaires with the help of their parent/carer (Supplemental Table 2).

We will measure children's height and weight from which we will calculate each child's body mass index (BMI) as weight/height^2 (kg/m^2) and standardized by age and sex.

Covariates

Parental physical and mental health is a strong predictor of child functioning.[40] We will therefore collect parental self-reported physical activity, mental health, wellbeing and general health. The International Physical Activity Questionnaire (IPAQ) – Short Form[41] will measure time spent in different intensities of physical activity during the last 7 days. We will use the Warwick and Edinburgh Mental Wellbeing Scale (WEMWBS) which includes fourteen items of feeling and functioning aspects of wellbeing to measure parental mental health.[42]

For peer review only

For peer review only - http://bmjopen.bmj.com/site/about/guidelines.xhtml

We will collect socio-demographic data on children's date of birth, sex, and ethnic group, and parental socio-demographic data including age, sex, ethnic group, highest level of qualification and employment status. These data will be collected through questionnaires completed by the parent/carer. For the covariates of age/date of birth, sex and ethnic group of the children and parent/carer, the questions will only be included at baseline. Parent/Carer qualification and employment data will be asked at each follow-up stage as these could change throughout the study period.

School characteristics including school size (i.e., overall pupil numbers), class size, ethnic composition, area level of deprivation and number of pupils eligible for free school meals will be obtained from publicly available data provided by the Department for Education. School participation in TDM will be sought at the start of each follow-up data collection.

Data collection

We will email a link to the questionnaires to parent/carers and class teachers. Paper versions will be available on request. Parents will complete the child questionnaires by proxy.

The iMprOVE research team will arrange a convenient time to visit the school to take anthropometry measures of the children and issue and fit them with the accelerometer. Verbal instructions of wearing the monitor will be given along with an information pack including details of wearing the monitor correctly, how long to wear, and when and how to return the monitors. Incentives (shopping voucher) will be offered to the child to encourage successful completion of all their corresponding data collection.

All measures taken at baseline will be repeated at each follow-up. From Wave 3 onwards, the children (now in Year Group 3 and aged 7 to 8 years) will be encouraged to complete their own questionnaires with help from their parent/carer. Incentives for the children after each wave of data collection will be offered to encourage families to continue taking part.

Data collection time points

There will be one data collection in each school year. Data collection for baseline (Wave 1) will begin from January 2021 (allowing for regular TDM activity to take place if implemented by the school) and will continue until we have met our sample size targets. Control group participants will be assessed simultaneously to the intervention group (Figure 1).

For consistency and potential seasonal variation in activity levels, we aim to carry out follow-up assessments during the same month as baseline assessments in each year until the end of data collection in 2026 (Figure 1).

Sample size

Our sample size is based on the primary outcome of mean MVPA (minutes) per day allowing for the clustering of children in the same school and the primary analysis adjusting for baseline minutes of MVPA. Based on our previous work, there is an average of 46 year 1 children per state-funded primary school in Greater London.[11]

We conducted a sample size calculation (using Stata v15.1) that produced a total sample of 3,533 children (approximately 1,767 children in the intervention and control groups) across 77 schools to give the study 90% power to detect a 5.5 minute increase in the mean daily average MVPA minutes between

For peer review only - http://bmjopen.bmj.com/site/about/guidelines.xhtml

children in TDM schools compared with non-TDM schools, assuming a mean daily average MVPA of 53 minutes (standard deviation 22) in the control group based on the study by Chesham et al.[15] This has been inflated to allow for 60% attrition across the follow-up period and assumes a two-sided alpha equal to 0.05. To allow for the analyses to include clustering at school level, we have applied an intraclass correlation coefficient of 0.05 to our calculation (Supplemental Table 3). An increase of just over 5 minutes of MVPA per day equal to a 10% difference would be clinically significant to increase physical activity among the proportion of children who participate in less than an average of 30 minutes per day.

Statistical analyses

For every wave of data collection, the main analysis will treat the outcomes as continuous variables. This will enable us to understand the impact of TDM in any change observed in physical activity, mental health and educational attainment for each child through each successive year of primary school.

We will use a linear multilevel mixed effect regression model to estimate the mean difference in MVPA values between TDM schools and non-TDM schools across follow-up and at each follow-up wave. Children will be included as a random intercept with fixed effects for wave, intervention group, wave-by-intervention group interaction and baseline (Wave 1) MVPA. School will also be included in the model as a random effect and adjustments for covariates including participation in TDM and socio-demographic variables will be included. Baseline adjusted group differences will be reported with a 95% confidence interval for each wave. An additional model without wave-by-intervention interaction will be fitted to provide an estimate of the overall intervention effect across follow-up.

Based on previous studies,[40, 43] we expect data from children to vary throughout each wave of data collection as compliance in the youngest age groups (5 to 6 years) may differ compared with when they are measured later (aged 10 to 11 years). Decisions on valid accelerometer data to include in the analysis will be made when reviewing the data and based on previous literature.

Linear multilevel mixed effect regression models as described for MVPA will be repeated for the outcomes of mental health and educational attainment.

Sub-group analysis

To address inequalities, we will examine MVPA of children in TDM and non-TDM schools according to area level deprivation (a measure of poverty) using the Index of Multiple Deprivation[44].

Sensitivity analyses

We anticipate that during the study period, schools that are assigned to intervention group may stop TDM, and schools assigned to the control group may take up TDM. We will conduct a series of sensitivity analyses to explore TDM impacts in children who engage in TDM in some year groups only. We will also consider seasonal effects on implementation of TDM and MVPA overall dependent on the month children are assessed. In addition, missing data for MVPA in the follow-up waves will be imputed and included separately to a complete case analysis.

All analyses will be carried out using STATA v15 (StataCorp, Texas, USA).

For peer review only - http://bmjopen.bmj.com/site/about/guidelines.xhtml

Patient and public involvement

We will conduct focus group workshops with children in the intervention group to ask about their views on taking part in TDM activities and their physical activity inside and outside of school. A separate focus group workshop will be conducted with the control group to obtain their views on physical activity inside and outside of school. The workshops will take place once a year during each year of data collection.

We have consulted professional and public representatives within the management structure (The Daily Mile Research Advisory Group including members from The Daily Mile Foundation, academic researchers, and representatives from Sport England, London Marathon and London Sport, and Patient and Public Involvement including parents and children who have been approached for piloting questionnaires and assessments) throughout the design of the study and who will continue to be consulted throughout the duration of the study. Advice on the design of study materials (e.g., study information sheets and questionnaires), participant engagement, and how to retain participants in the study will be sought throughout the study period. Questionnaires will be piloted with parents and children through workshops.

Discussion

Currently, physical activity has fallen to lowest rates following the Covid-19 pandemic lockdowns and restrictions. Global policies are focusing on safe ways that will allow children to adapt to primary school life that allows improved health behaviours aimed at increasing physical activity. Active mile initiatives in school settings are seen as part of the solution to rebuilding and embedding health behaviours in early life.

The iMprOVE study will be the most comprehensive evaluation of an active mile initiative and the first longitudinal study to capture the impact of participation over primary school life.

Key strengths of the iMprOVE study are its size, the assessment of an intervention in a real world setting, and individual level data and longitudinal follow-up throughout primary school life. Furthermore, the objective measure of physical activity using accelerometers will provide more accurate estimates of daily minutes of MVPA than parent-reported physical activity in questionnaires, minimising potential participant bias of overestimating or underestimating self-reported time spent being active.[45] The inclusion of schools from a diverse multi-ethnic population and urban setting enables us to examine differential impacts of TDM among different socio-demographic groups. However, there are a number of important limitations to consider. Since iMprOVE is a quasi-experimental cohort study and not randomised, there is likely to be an unequal number of participants in the intervention and control groups. We therefore expect some selection bias in schools that agree to participate in the study and as with previous cohort studies, there may be underrepresentation of school populations from disadvantaged areas.

Given the popularity of TDM and its widescale adoption across the UK and globally, the results of this study will be of interest to parents, teachers and public health policy makers. The findings will determine the potential of TDM and other similar active mile initiatives, to improve the health, wellbeing and

For peer review only - http://bmjopen.bmj.com/site/about/guidelines.xhtml

educational attainment for all children in primary schools and inform national strategies to reduce inequalities in child health.

Ethics and dissemination

Full ethical approval has been granted by Imperial College Research Ethics Committee (ICREC), reference 20IC6127.

Wider dissemination of key findings to the public will be through research networks, social (Facebook, Twitter), print (newspapers) and media broadcasts (news outlets). We will engage with study participants through community engagement activities, presentations, and newsletters to schools, teachers, parents and children taking part in the study. We will work with policy makers nationally to ensure direct application and impact of our work.

For peer review only

Project steering

The management structure of this study includes the following groups:

1. Investigators (Principal Investigator and Lead Researcher)
2. Research support team (collaborators)
3. Project steering group (advisors)

Author contributions

SS wrote the original application and obtained funding for the study. BR and SS conceptualized the cohort study. BR, SS, AC, EvS, DH and RV designed the study. BR and RP developed the statistical analysis plan. BR wrote the methodology and developed all the study materials. BR and SS wrote the ethics application and gained ethical approval from Imperial College Research Ethics Committee (ICREC). BR produced the first draft of this manuscript. BR, SS, AC EvS DH RV and TV contributed to redrafts.

Funding statement

This work is funded by The Daily Mile Foundation supported by INEOS.

BR is funded by The Daily Mile Foundation. SS is supported by The Daily Mile Foundation, the National Institute for Health Research (NIHR) School for Public Health Research (SPHR) and the NIHR North West London Applied Research Collaboration (NW London ARC). TV is funded by an NIHR SPHR PhD Studentship (Grant Reference Number PD-SPH-2015-10055). DH is part of the NIHR SPHR and NW London ARC, and supported by the Department for Education (DfE).

EvS is supported by the Medical Research Council (MRC) (Grant MC_UU_12015/7). The work by EvS is undertaken under the auspices of the Centre for Diet and Activity Research (CEDAR), where funding from Cancer Research UK, the British Heart Foundation, the Economic and Social Research Council, the Medical Research Council, the National Institute for Health Research, and the Wellcome Trust, under the auspices of the UK Clinical Research Collaboration, is gratefully acknowledged (087636/Z/08/Z; ES/G0007462/1; MR/K023187/1).

Acknowledgements

We are grateful for support from the NIHR School for Public Health Research and the NIHR North West NW London ARC.

The NIHR School for Public Health Research is a partnership between the Universities of: Sheffield; Bristol; Cambridge; Imperial; and University College London; The London School for Hygiene and Tropical Medicine (LSHTM); LiLaC – a collaboration between the Universities of Liverpool and Lancaster; and Fuse - The Centre for Translational Research in Public Health a collaboration between Newcastle, Durham, Northumbria, Sunderland and Teesside Universities.

The Department of Primary Care and Public Health is grateful for support from the NIHR Biomedical Research Centre funding scheme, the NIHR School for Public Health Research, and the NIHR North West London Applied Research Collaboration.

We thank The Daily Mile Foundation for funding and support.

For peer review only

For peer review only - http://bmjopen.bmj.com/site/about/guidelines.xhtml

The views expressed here are those of the authors and not necessarily those of the NIHR, the Department of Health and Social Care, the NHS, DfE or The Daily Mile Foundation.

Competing interests

SS and BR received funding from The Daily Mile Foundation. SS, BR, AC and TV are members of The Daily Mile Research Advisory Group.

Patient consent for publication

Not required.

Data sharing statement

Data sharing is not applicable to this protocol. Data collected as part of this research will be available to external researchers on completion of the study, and proposals for collaboration prior to, during and after study completion are welcome.

Open access

This is an open access article distributed in accordance with the Creative Commons Attribution Non Commercial (CC BY-NC 4.0) license, which permits others to distribute, remix, adapt, build upon this work non-commercially, and license their derivative works on different terms, provided the original work is properly cited, appropriate credit is given, any changes made indicated, and the use is non-commercial. See: http:// creativecommons. org/ licenses/ by- nc/ 4. 0/.

Figures

Figure 1 iMprOVE study design.

Figure 2 Identifying schools to be included in the iMprOVE study.

For peer review only

For peer review only - http://bmjopen.bmj.com/site/about/guidelines.xhtml

References

1. WHO. Global recommendations on physical activity for health. Geneva: World Health Organisation; 2010.

2. WHO. Global Health Risks: Mortality and burden of disease attributable to selected major risks. Geneva: World Health Organisation; 2009.

3. World Health Organisation. Global Strategy on Diet, Physical Activity and Health; Physical activity and young people Geneva2011 [Available from: https://www.who.int/dietphysicalactivity/factsheet_young_people/en/ [Accessed Jul 2020].

4. NCMP. National Child Measurement Programme (NCMP), England 2018/19 School Year. United Kingdom: National Statistics, NHS Digital; 2019.

5. Department of Health and Social Care. Physical activity guidelines: UK Chief Medical Officers' Report. GOV.UK; 2019.

6. Sport England. Active Lives: Children and Young People Survey, Academic Year 2018/19. UK; 2019.

7. Public Health England. Active mile briefing: evidence and policy summary. United Kingdom: Loughborough University, UK, for Public Health England; 2020.

8. Waters E, de Silva-Sanigorski A, Hall BJ, et al. Interventions for preventing obesity in children. Cochrane Database Syst Rev. 2011(12):CD001871.

9. Wang Y, Cai L, Wu Y, et al. What childhood obesity prevention programmes work? A systematic review and meta-analysis. Obes Rev. 2015;16(7):547-65.

10. The Daily Mile Foundation. The Daily Mile [Available from: https://thedailymile.co.uk/.

11. Venkatraman T, Honeyford K, Costelloe C, et al. Sociodemographic profiles, educational attainment and physical activity associated with The Daily Mile registration in primary schools in England – a national cross-sectional linkage study. Journal of Epidemiology and Community Health. 2020(0):1-8.

12. The Daily Mile UK. The Daily Mile Global Map United Kingdom2019 [Available from: https://thedailymile.co.uk/participation-map/ [Accessed March 2020].

13. HM Government. Childhood obesity: a plan for action Chapter 2. United Kingdom; 2018.

For peer review only

14. Marmot M, Allen J, Boyce T, et al. Health equity in England: The Marmot Review 10 years on. London: Institute of Health Equity; 2020.

15. Chesham RA, Booth JN, Sweeney EL, et al. The Daily Mile makes primary school children more active, less sedentary and improves their fitness and body composition: a quasi-experimental pilot study. BMC Medicine. 2018;16(1):64.

16. Morris JL, Daly-Smith A, Archbold VS, et al. The Daily Mile (TM) initiative: Exploring physical activity and the acute effects on executive function and academic performance in primary school children. Psychol Sport Exerc. 2019;45.

17. Chalkley AE, Routen AC, Harris JP, et al. "I Just Like the Feeling of It, Outside Being Active": Pupils' Experiences of a School-Based Running Program, a Qualitative Study. J Sport Exercise Psy. 2020;42(1):48-58.

18. Marchant E, Todd C, Stratton G, et al. The Daily Mile: Whole-school recommendations for implementation and sustainability. A mixed-methods study. PLoS One. 2020;15(2):e0228149.

19. Booth JN, Chesham RA, Brooks NE, et al. A citizen science study of short physical activity breaks at school: improvements in cognition and wellbeing with self-paced activity. BMC Med. 2020;18(1):62.

20. Breheny K, Passmore S, Adab P, et al. Effectiveness and cost-effectiveness of The Daily Mile on childhood weight outcomes and wellbeing: a cluster randomised controlled trial. Int J Obesity. 2020;44(4):812-22.

21. Wareham NJ, van Sluijs EM, Ekelund U. Physical activity and obesity prevention: a review of the current evidence. Proc Nutr Soc. 2005;64(2):229-47.

22. Hanckel B, Ruta D, Scott G, et al. The Daily Mile as a public health intervention: a rapid ethnographic assessment of uptake and implementation in South London, UK. BMC Public Health. 2019;19(1):1167.

23. Malden S, Doi L. The Daily Mile: teachers' perspectives of the barriers and facilitators to the delivery of a school-based physical activity intervention. BMJ Open. 2019;9(3):e027169.

24. Ryde GC, Booth JN, Brooks NE, et al. The Daily Mile: What factors are associated with its implementation success? PLoS One. 2018;13(10):e0204988.

25. The Daily Mile UK. The Daily Mile Core Principles United Kingdom2019 [Available from: https://thedailymile.co.uk/steps-to-success [Accessed March 2020].

For peer review only

For peer review only - http://bmjopen.bmj.com/site/about/guidelines.xhtml

483 26. Brown HE, Whittle F, Jong ST, et al. A cluster randomised controlled trial to evaluate the effectiveness and cost-effectiveness of the GoActive intervention to increase physical activity among adolescents aged 13-14 years. Bmj Open. 2017;7(9).

486 27. Collings PJ, Wijndaele K, Corder K, et al. Levels and patterns of objectively-measured physical activity volume and intensity distribution in UK adolescents: the ROOTS study. Int J Behav Nutr Phy. 2014;11.

489 28. Lloyd J, Creanor S, Logan S, et al. Effectiveness of the Healthy Lifestyles Programme (HeLP) to prevent obesity in UK primary-school children: a cluster randomised controlled trial. Lancet Child Adolesc. 2018;2(1):35-45.

492 29. Duncan MJ, Wilson S, Tallis J, et al. Validation of the Phillips et al. GENEActiv accelerometer wrist cut-points in children aged 5-8 years old. Eur J Pediatr. 2016;175(12):2019-21.

494 30. Goodman R. The Strengths and Difficulties Questionnaire: a research note. J Child Psychol Psychiatry. 1997;38(5):581-6.

496 31. Muris P, Meesters C, van den Berg F. The Strengths and Difficulties Questionnaire (SDQ)--further evidence for its reliability and validity in a community sample of Dutch children and adolescents. Eur Child Adolesc Psychiatry. 2003;12(1):1-8.

499 32. CORC. Strengths and Difficulties Questionnaire (SDQ) London, UK: Child Outcomes Research Consortium; 2002 [Available from: https://www.corc.uk.net/outcome-experience-measures/strengths-and-difficulties-questionnaire/ [Accessed Jul 2019].

502 33. NFER. Understanding age-related expectations UK: The National Foundation for Educational Research in England and Wales; 2002-2018 [Available from: https://www.nfer.ac.uk/for-schools/free-resources-advice/assessment-hub/introduction-to-assessment/understanding-age-related-expectations/ [Accessed Jul 2020].

506 34. Kowalski K, Crocker R, Donen R. The Physical Activity Questionnaire for Older Children (PAQ-C) and Adolescents (PAQ-Q) Manual. Canada: University of Saskatchewan; 2004.

508 35. Stevens KJ. Working with children to develop dimensions for a preference-based, generic, pediatric, health-related quality-of-life measure. Qual Health Res. 2010;20(3):340-51.

510 36. Stevens K. CHU-9D: Measuring health and calculating QALYs for children and adolescents UK: The University of Sheffield; 2016 [Available from: https://licensing.sheffield.ac.uk/i/health-outcomes/CHU-9D.html [Accessed Jan 2020].

513 37. Stevens K. Valuation of the Child Health Utility 9D Index. Pharmacoeconomics. 2012;30(8):729-47.

For peer review only

For peer review only - http://bmjopen.bmj.com/site/about/guidelines.xhtml

38. Stevens K, Ratcliffe J. Measuring and valuing health benefits for economic evaluation in adolescence: an assessment of the practicality and validity of the child health utility 9D in the Australian adolescent population. Value Health. 2012;15(8):1092-9.

39. Petersen KD, Ratcliffe J, Chen G, et al. The construct validity of the Child Health Utility 9D-DK instrument. Health Qual Life Outcomes. 2019;17(1):187.

40. Jago R, Solomon-Moore E, Toumpakari Z, et al. Parents' perspectives of change in child physical activity & screen-viewing between Y1 (5-6) & Y4 (8-9) of primary school: implications for behaviour change. BMC Public Health. 2018;18(1):520.

41. Craig CL, Marshall AL, Sjostrom M, et al. International physical activity questionnaire: 12-country reliability and validity. Med Sci Sports Exerc. 2003;35(8):1381-95.

42. Tennant R, Hiller L, Fishwick R, et al. The Warwick-Edinburgh Mental Well-being Scale (WEMWBS): development and UK validation. Health Qual Life Outcomes. 2007;5:63.

43. Jago R, Salway R, Emm-Collison L, et al. Association of BMI category with change in children's physical activity between ages 6 and 11 years: a longitudinal study. Int J Obes (Lond). 2020;44(1):104-13.

44. Ministry of Housing CaLG. English Indices of Multiple Deprivation London, UK: Natiional Statistics; 2019 [Available from: https://www.gov.uk/government/statistics/english-indices-of-deprivation-2019 [Accessed June 2020].

45. DAPA measurement toolkit. [Available from: https://dapa-toolkit.mrc.ac.uk.

For peer review only - http://bmjopen.bmj.com/site/about/guidelines.xhtml

Figure 1. iMprOVE study design

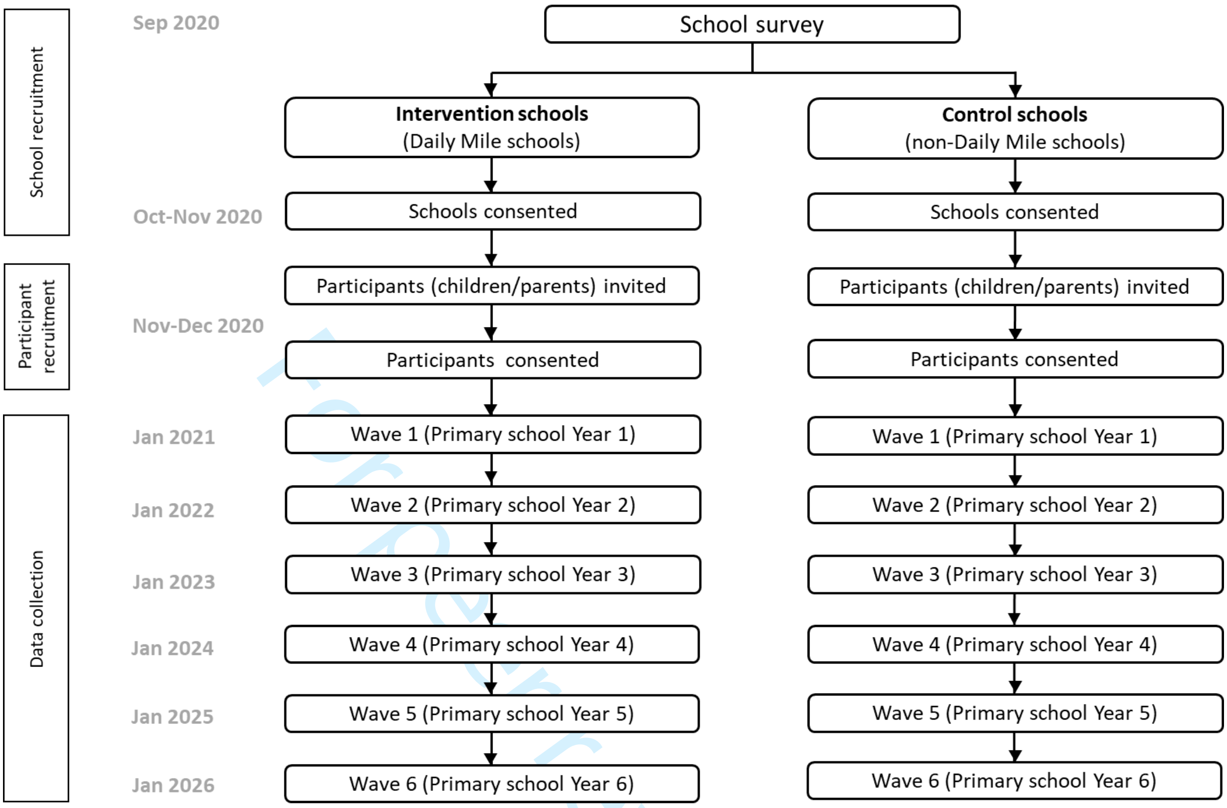

For peer review only

Figure 2. Identifying schools to be included in the iMprOVE study

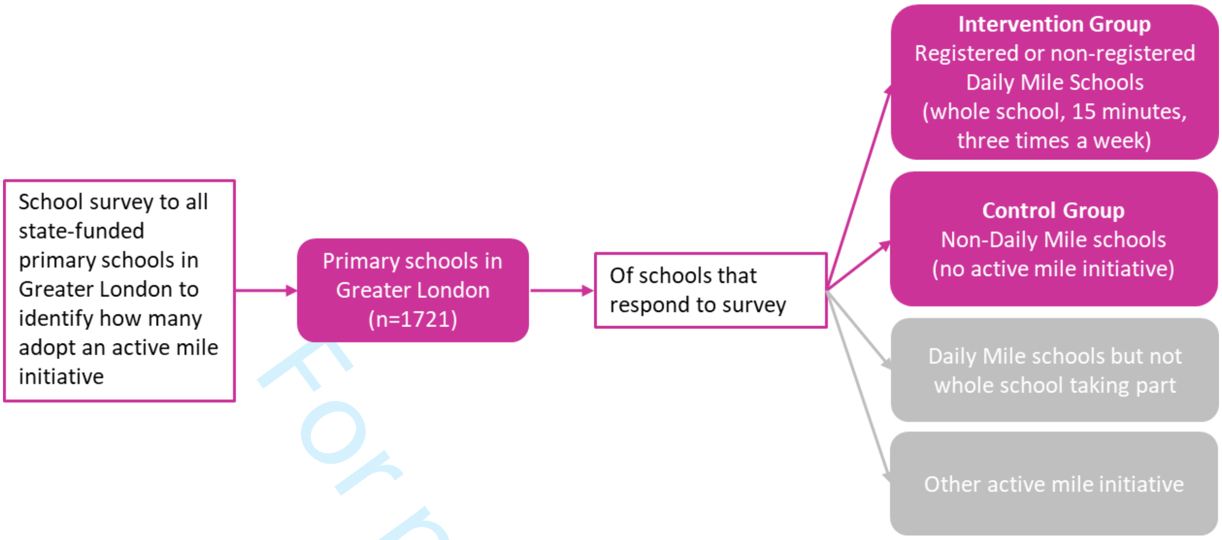

For peer review only

Supplemental Table 1. The Daily Mile's ten core principles[1]

Principle		Definition
1.	Quick	Just 15 minutes with no time spent changing, setting up or tidying up. Transitions between class and route should be slick.
2.	Fun	The Daily Mile is physical activity in a social setting and must be fun for the children. They can chat to their friends as they run along enjoying the experience together.
3.	100%	It's always fully inclusive – every child, every day. They should all be out together in the fresh air. Children with mobility difficulties should be supported to take part.
4.	Weather	Treat the weather as a benefit, not a barrier. Children enjoy being outside in the different types of weather, connecting with nature and being aware of the seasons.
5.	Route	Ideally, your Daily Mile route should have a firm and mud-free surface – most schools use the playground or an existing path. Incorporating child-pleasing loops and squiggles works well.
6.	Risk	Risk assess the route in order to ensure The Daily Mile is a safe activity.
7.	When to Go	The Daily Mile should happen during curricular time, at least 3 times a week. Ideally, the class teacher should decide when to go out – they know their class and can respond flexibly to their needs.
8.	Clothes	The children run in their school clothes without changing into kit, putting jackets on if it's cold or damp and taking sweatshirts off if it's warm.
9.	Own Pace	The children go at their own pace. Done properly, it's not a walk – able-bodied children should aim to run or jog for the full 15 minutes with only occasional stops to catch their breath, if necessary.
10.	Simple	Keep it simple. Resist the temptation to over complicate it. It should always be social and fun. From time-to-time, you may wish to connect it to the curriculum or do something seasonal, for example, running 'Laps to Lapland'.

[1]*The Daily Mile Foundation (https://thedailymile.co.uk/steps-to-success/)*

For peer review only - http://bmjopen.bmj.com/site/about/guidelines.xhtml

Supplemental Table 2. Outcomes and measuring tools included in the iMprOVE study

Outcome	Measured by	Outcome/completed by
Physical activity	• Device-based (accelerometer)<br>• Children's Physical Activity Questionnaire (C-PAQ)[a] Parent<br>• International Physical Activity Questionnaire (IPAQ)[b]	Children's physical activity<br>Parent/Carer to complete about their child's physical activity<br>Parent/Carer's* physical activity
Mental health	• The Warwick and Edinburgh Mental Wellbeing Scale (WEMWBS)[c]<br>• Strengths and Difficulties Questionnaire (SDQ)[e]	Parent/Carer's* mental health<br>Parent/Carer's* and Teachers to complete about the child
Educational attainment	• Likert scale from 1 (below expected levels) to 5 (above expected levels)[f]	Teacher's ratings
General health and wellbeing	• Child Health Utility 9D (CHU-9D)[d]<br>• EuroQol Visual Analogue Scale (EuroQol VAS)[g]<br>• EuroQol Descriptive Scale[g]<br>• Questions from the UK Census 2011[h]	Parent/Carers to complete for their children in school years 1 and 2. For children in years 3 to 6, children will be encouraged to complete questions by themselves with help from parent/carer.<br>Parent/Carer's general health, and parent/carer to complete for their child in school years 1 and 2. Children in years 3 to 6, children will be encouraged to complete questions by themselves with help from parent/carer.<br>Parent/Carer general health<br>Parent/Carer general health, and parent/carer to complete for their child in school years 1 and 2. Children in years 3 to 6, children will be encouraged to complete questions by themselves with help from parent/carer.

For peer review only

	• Life satisfaction, worthwhile, happiness and anxiety questions from Measuring National Wellbeing (MNW)[i]	Parent/Carer wellbeing
Socio-demographic	• Questions from the UK Census 2011[h]	Parent/Carer's own socio-demographic data, and parent/carer to complete socio-demographic data about their child if their child is in school years 1 or 2. Children in years 3 to 5 will be encouraged to complete these questions on their own with help from their parent/career.
Anthropometry	• Height measure<br>• Bioimpedance scales	Children's height and weight.

*Any parent/carer for the child can complete the questionnaires at baseline. For consistency, we will ask the parent/carer who completed the questionnaire at baseline to complete questionnaires at each follow-up.

[a]Kowalski k, Crocker R and Donen R. The Physical Activity Questionnaire for Older Children (PAQ-C) and Adolescents (PAQ-Q) Manual. University of Saskatchewan.

[b]Craig, C. L., et al. (2003). "International physical activity questionnaire: 12-country reliability and validity." Med Sci Sports Exerc 35: 1381-95.

[c]Tenant R, Hiller L, Fishwick R et al. The Warwick-Edinburgh Mental Well-being Scale (WEMWBS): development and UK validation. *Health Qual Life Outcomes.* 2007;5. DOI: 10.1186/1477-7525-5-63.

[d]Stevens KJ. Working with Children to Develop Dimensions for a Preference-Based, Generic, Paediatric, Health-Related Quality-of-Life Measure. *Qual Health Res.* 2010;20(3):340-351. DOI: 10.1177/1049732309358328.

[e]Goodman R. The Strengths and Difficulties Questionnaire: a research note. *Journal of Child Psychology and Psychiatry.* 1997;38(5):581-586. DOI: 10.1111/j.1469-7610.1997.tb01545.x.

[f]Breheny K, Passmore S, Adab P, et al. Effectiveness and cost-effectiveness of The Daily Mile on childhood weight outcomes and wellbeing: a cluster randomised controlled trial. *International Journal of Obesity.* 2020;44(4):812-822. DOI: 10.1038/s41366-019-0511-0.

[g]EuroQol Research Foundation. EQ-5D-5L User Guide 2019.

[h]UK Census United Kingdom. Office for National Statistics (ONS). 2011.

[i]Measuring National Wellbeing – Personal Wellbeing. Office for National Statistics, 2019

For peer review only

Supplemental Table 3. iMprOVE study sample size including clustering at school level, Intraclass Correlation (ICC) of 0.05, and 60% attrition

Unit (cluster) level	School
Clusters	77
Cluster size (pupils)	46
Allocation ratio	1:1
Control group MVPA (mins)	53
Intervention group MVPA (mins)	58.5
Variance MVPA	484
Standard deviation MVPA	22
Power	0.9
Alpha (two-sided)	0.05
ICC[1]	0.05
Total sample size (without inflation)	2208
Drop out	60
Extra pupils	1325
Total sample size (inflated for drop-out)	3533
**Sample size per group**	**1766**

[1]ICC: Intraclass correlation

For peer review only
